# Systems and Methods for Transformation and Degradation Analysis

**DOI:** 10.3390/e26060454

**Published:** 2024-05-27

**Authors:** Jude A. Osara, Michael D. Bryant

**Affiliations:** 1 Surface Technology and Tribology, Department of Mechanics of Solids, Surfaces and Systems, University of Twente, 7522 NB Enschede, The Netherlands; 2 Mechanical Engineering Department, University of Texas at Austin, Austin, TX 78712, USA; michael.bryant@machineessence.com; 3 Machine Essence Corporation, Austin, TX 78746, USA

**Keywords:** systems analysis, aging, degradation analysis, transformation analysis, entropy generation, temperature, degradation thermodynamics, transformation thermodynamics, hyperplane, trajectory, second law, PEG, DEG, TPEG

## Abstract

Modern concepts in irreversible thermodynamics are applied to system transformation and degradation analyses. Phenomenological entropy generation (PEG) theorem is combined with the Degradation-Entropy Generation (DEG) theorem for instantaneous multi-disciplinary, multi-scale, multi-component system characterization. A transformation-PEG theorem and space materialize with system and process defining elements and dimensions. The near-100% accurate, consistent results and features in recent publications demonstrating and applying the new TPEG methods to frictional wear, grease aging, electrochemical power system cycling—including lithium-ion battery thermal runaway—metal fatigue loading and pump flow are collated herein, demonstrating the practicality of the new and universal PEG theorem and the predictive power of models that combine and utilize both theorems. The methodology is useful for design, analysis, prognostics, diagnostics, maintenance and optimization.

## 1. Introduction

To exist is to degrade. Degradation occurs everywhere and in everything. The performance, utility and reliability of engineering systems depend on how fast they age over time. The preventive maintenance of systems susceptible to catastrophic failure costs billions of dollars annually. For example, transportation systems—aircraft, automotives, railway systems—are often prematurely serviced at regular intervals irrespective of the actual health of the system. There remains an urgent need for improved degradation characterization and failure prediction methods for maintenance optimization and failure prevention.

Dissipation via forces and processes, such as friction, plasticity, chemical reactions, dislocation movements and corrosion, leads to system or component failure. There are countless examples in human daily experiences, a few with industrial ramifications, including frictional damage in tribological interfaces, capacity fade and thermal instability in batteries, mechanical fatigue of metallic structures and interfaces, thermal fatigue of electrical/electronic components and lubricant degradation (leading to bearing and machine failures), among others. Currently, manufacturers rely on expensive heuristics: empirical models requiring extensive data and statistical analyses to estimate system degradation and predict a component’s remaining useful life. System maintenance and optimization rely on resource-heavy artificial intelligence techniques. In addition to being inconsistent, these models often cannot be adapted to similar systems without significant corrections.

Material/system formation, opposite to degradation, transforms materials into more useful or higher-energy forms. Examples are raw materials that, via a series of manufacturing processes, transform into finished products [1], such as a discharged battery that recharges and is available for re-use. Rechargeable systems such as batteries do not degrade monotonically, with the recharge step restoring the battery’s health over some cycles. Several materials recover/heal when constraints are removed or reversed. Real transformations, whether positive (formation/healing) or negative (degradation), involve energy conversions accompanied by losses, as per experience and the second law of thermodynamics. Optimum transformation and minimum degradation are desired. This article develops a methodology for evaluating the instantaneous transformation of any system via the system’s phenomenology.

Most transformation/degradation models are system- and discipline-specific. Transformation or degradation often involves many different mechanisms, some of which are concurrent. The first and second laws of thermodynamics govern energy transformation—conversion and dissipation—in real (*irreversible*) systems. Recent advances in the thermodynamics of real systems—*irreversible thermodynamics*—have demonstrated applicability to degradation characterization [2]. Lemaitre and Chaboche [3] combined continuum mechanics with irreversible thermodynamics to develop widely used constitutive relations that characterize dissipation in solid mechanics. Bejan [4,5] optimized thermal systems by minimizing entropy generation. Kuhn [6,7] defined a rheological energy density to describe grease transformation due to imposed shear energy. Khonsari et al. [8,9,10,11,12,13,14] applied *single-variable* entropy generation to characterize the degradation of materials such as metals, composites and lubricant grease; and mechanisms such as frictional wear. Sosnovskiy and Sherbakov [15,16], via various studies, characterized complex, simultaneously occurring damage mechanisms by correlating a damage variable to single-variable entropy generations. Basaran et al. [17,18,19] combined aspects of Boltzmann’s entropy and Newtonian mechanics to define a Unified Mechanics Theory with demonstrated success in failure prediction. These and several others have presented accurate real-world results, with shortcomings attributable to assumptions. Osara and Bryant [20,21,22,23,24,25,26] directly correlated phenomenological entropy generation (PEG) [27] with a transformation/degradation measure, as prescribed by the degradation–entropy generation (DEG) theorem [28], to describe highly dissipative system–process interactions at near-100% accuracy.

In this treatise, the transformation dynamics of the PEG theorem are combined with the degradation dynamics of the DEG theorem to render a **transformation–phenomenological entropy generation (TPEG)** theorem with practical use in the design, health/performance monitoring, diagnostics and optimization of all macroscopic systems. We establish a direct relationship between the instantaneous transformation (and degradation) of systems undergoing *irreversible* processes—real processes including multiple concurrent, high-rate, anisothermal processes—and the accompanying phenomenological entropy generation. Example applications for several multi-disciplinary engineering systems are demonstrated.

## 2. Definitions

The following definitions are relevant to the discussions in this article:

1. *Observable:* a metric such as a physical property or performance indicator is observable if it can be sensed and measured directly.
2. *Phenomenological:* characterized by observable phenomena, such as volume expansion.
3. *Transformation:* a change in state quantified by the difference between the instantaneously observable time varying value of a **non-monotonic** transformation measure (or performance indicator) and its initial/reference value.
4. *Phenomenological transformation/degradation* $w_{phen}$*:* the instantaneous transformation/degradation of a system or material via a **non-monotonic** transformation/degradation measure (or performance indicator).
5. *Phenomenological entropy generation* $S_{phen}$*:* the instantaneous entropy generation along the transformation path through state space *Z*, observable through the state variables that characterize the active interactions, is always the sum of all active work and compositional change entropy generations and MST entropy [27]. Unlike entropy generation *S′*, which is always non-negative, $S_{phen}$ is positive for energy addition and negative for energy extraction, in accordance with IUPAC sign convention.
6. *Reversible transformation* $w_{rev}$*:* the idealized, quasi-static transformation of a system or material. $w_{rev}$ can be estimated as the healthiest state transformation.

## 3. A Review of the Degradation–Entropy Generation Theorem

A quantitative study of system degradation by active dissipative processes formulated the degradation–entropy generation (DEG) theorem [28], establishing a direct relationship between monotonic degradation *w* and the irreversible entropies ${S^{\prime }}_{i}$ generated by the dissipative processes.

### Statement

Given irreversible material degradation consisting of *i* = 1, 2, …, *n* dissipative processes $p_{i}$, which could describe an energy, work, or heat characteristic of the process. Assume the effects of the mechanism can be described by a parameter or state variable *w* that measures the effects of the degradation, such that $w=w\left(p_{i}\right)=w\left(p_{1},p_{2},\ldots ,p_{n}\right),i=1,\;2,\ldots ,n$ is monotonic in each $p_{i}$. Then, the rate of change in the degradation

(1) $$\dot{w}=\sum_{i}B_{i}{\dot{S}\prime }_{i}$$

is a linear combination of the rates of entropies ${\dot{S}\prime }_{i}$ generated by the dissipative processes $p_{i}$, where the degradation coefficients

(2) $$B_{i}={\left.\frac{\partial w}{{\partial S^{\prime }}_{i}}\right|}_{p_{i}}$$

are the slopes of degradation *w*, with respect to entropy generation ${S^{\prime }}_{i}$; the subscript notation ${\;}_{p_{i}}$ indicates that process $p_{i}$ is active. Integrating Equation (1) over time *t* yields the cumulative degradation

(3) $$w=\sum_{i}B_{i}{S^{\prime }}_{i}$$

a linear combination of the accumulated entropies ${S^{\prime }}_{i}$ generated by the dissipative processes $p_{i}$. Details of the DEG theorem, including statements and proof, can be found in [28].

## 4. A Review of the Phenomenological Entropy Generation Theorem

A first principles combination of the thermodynamic potentials with the first and second laws of thermodynamics formulated the phenomenological entropy generation (DEG) theorem [28], describing the instantaneously observable entropy generation of any real system undergoing active processes.

### 4.1. Statement

For any transforming system undergoing energy changes, entropy generation is the difference

(4) $$\delta S\prime ={\delta S}_{phen}-{dS}_{rev}\geq 0$$

between a phenomenological entropy generation function ${\delta S}_{phen}$, evaluated via suitable time-based measurements or estimates of system and process variables between initial and final (or current) thermodynamic states and a reversible entropy function ${dS}_{rev}$, evaluated via end state measurements of system variables (before and after process interactions). To maintain the non-negative inequality of Equation (4) mandated by the second law, for energy extraction or system loading, ${dS}_{rev}\leq {\delta S}_{phen}<0$, and for energy addition or system formation, $0<{dS}_{rev}\leq {\delta S}_{phen}$. 

δS′ **quantifies energy dissipation/degradation while** ${\delta S}_{phen}$ **characterizes total energy transformation including conversion and dissipation.** Details of the PEG theorem, including statements and derivation, can be found in reference [27].

### 4.2. Corollary

According to the thermodynamic state postulate [29], *the state of a simple system is completely specified by r + 1 independent properties*, where *r* is the number of active independent work interactions. Hence, no real unsteady system can be fully defined by one interaction or property. Similarly, deriving from first principles, a corollary of the PEG theorem asserts the following [22]:

*Given an r number of active work interactions, the phenomenological entropy generation of a simple system is completely specified by r + 1 independent properties.*

Single-variable entropy generations ${\delta S\prime }_{W,r}=\sum_{r}\frac{Y_{r}dX_{r}}{T}$ [30] govern all the *r* active (internal and external) work and flow mechanisms, while MicroStructuroThermal (MST) entropy $\delta {S\prime }_{\mu T}=-\frac{SdT}{T}$ [23,24,27] governs the accompanying coupled microstructural and thermal mechanisms:

(5) $$\delta S_{phen}=\delta {S\prime }_{W,r}+\delta {S\prime }_{\mu T}.$$

Here, $\delta {S\prime }_{W,r}=\delta {S\prime }_{W}+d{S\prime }_{N}$, where $\delta {S\prime }_{W}$ represents all the boundary/external interactions (or work transfers), and $d{S\prime }_{N}$ is the internal compositional change (including matter flow) entropy. *Y* is generalized force, *dX* is generalized displacement, *dT* is the temperature change and *S* is the entropy content defined by material properties and process variables [27] as

(6) $$S_{A}=C_{X}lnT+\beta X-\lambda_{X}N\;\mathrm{and}\;S_{G}=C_{Y}lnT-X\alpha Y-\lambda_{Y}N$$

where $S_{A}$ is the Helmholtz-based entropy content, for boundary-loaded (work-capable) systems, and $S_{G}$ is the Gibbs-based entropy content, for internally reactive systems. $C_{X}$ and $C_{Y}$ are heat capacities, β=αE′ is the thermal stress/strength coefficient, α is the thermal strain coefficient, *E’* is the load modulus, $\lambda_{X}$ and $\lambda_{Y}$ are the thermal chemico-transport decay coefficients, *T* is temperature and *N* is the number of moles of active/reactive species. The MST energy −SdT is the system-stored portion of the free energy dissipated by mechanisms such as friction, plasticity and others, manifesting as the temperature rise of a *non-thermal* system [27]. According with the definition of entropy *S* as the measure of a system’s microstructural order, Equation (6) governs the microstructural changes in the system that drive the temperature rise *dT* in MST energy/entropy. The first term characterizes the thermal energy (which is directly related to the kinetic energy of the atoms and molecules). The second term characterizes the thermal expansion/strain effects and structural response to load. The third term involves the chemical (compositional change) response to temperature. In steady state, very slow and/or isothermal processes, dT≈0 to render $\delta {S\prime }_{\mu T}\approx 0$. For electrochemical systems wherein $\delta {S\prime }_{\mu T}$ is named ElectroChemicoThermal (ECT) entropy, via the Gibbs–Duhem formulation and Faraday’s electrolysis laws, $\delta {S\prime }_{\mu T}=qdv$ where q is charge content and v is potential difference or voltage [27].

Table 1 presents examples of $\delta {S\prime }_{W,r}$ for various active work mechanisms. In some situations, multiple processes could be active, for example during the wall ironing of sheet metal, involving both frictional work and tension–compression work. In such cases, $\delta {S\prime }_{W,r}$ is the sum of all the active single-variable entropy generations.

#### 4.2.1. Heat and Work Lines—Instantaneous Orthogonality

Heat and work are different manifestations of energy, represented with different terms in the energy balance. While an algebraic sum of both heat and work yields the internal energy change between two states when the end state values of the system variables are unknown, the sum does not give useful information on—for example, which mechanism dominates—the system transformation between both states. Hence, a heat line, perpendicular to a work line, was used in classical thermodynamics to correlate both forms of energy transformations. Here, in anticipation of the different contributions from each active mechanism to transformation, the phenomenological entropy components—work entropy ${S\prime }_{W,r}$ and MST entropy ${S\prime }_{\mu T}$—are orthogonal, introducing an *r+1* dimensional space through which the system’s phenomenological entropy traverses.

#### 4.2.2. Dissipation Factor *J* and Entropic Efficiency $\eta_{S\prime }$

The dissipation factor [21,27,31] which measures a non-thermal system’s dissipation tendencies relative to useful work output/chemical reaction is

(7) $$J=\frac{{S\prime }_{\mu T}}{{S\prime }_{W,r}}.$$

MicroStructuroThermal MST entropy measures the storage of free energy dissipation. Hence, a low *J* is desired for slow degradation and optimum performance/transformation.

The entropic efficiency [27] which measures the portion of the theoretical maximum work instantaneously obtained as real work/reaction is

(8) $$\eta_{S\prime }=\frac{{S\prime }_{W,r}}{S_{rev}}.$$

An ideal system—for which ${S\prime }_{W,r}=S_{rev}$—establishes 100% efficiency; hence, a high $\eta_{S\prime }$ is preferred for optimum performance and durability. Note that both non-dimensional factors *J* and $\eta_{S\prime }$ are associated with the relative slopes of phenomenological entropy trajectory through the *r + 1* dimensional space spanned by ${S\prime }_{W,r}$ and ${S\prime }_{\mu T}$.

## 5. Transformation–Phenomenological Entropy Generation Theorem

### 5.1. Reversible/Quasi-Static Transformation $w_{rev}$

Ideal/reversible/quasi-static transformation does not include active dissipative mechanisms. Here, the change in free energy is equal to the maximum work obtainable from or the minimum work addable to the system, serving as a theoretical reference asymptotically approached by extremely slow real processes. Hence, true reversible transformation, having no time considerations, is unobservable in a real system. However, established physics laws governing most physical phenomena can be used to estimate reversible transformation. Examples are Hooke’s law and Bernoulli’s equation. When such models are unavailable, a very slow process—occurring at the healthiest state of the system—with nearly constant intensive properties, such as temperature and pressure, can be used to estimate the reversible transformation.

### 5.2. Phenomenological Transformation $w_{phen}$

In most systems, readily accessible transformation measures are often related to the utility of the system and, hence, related to a primary form of loading or work. Temperature effects and effects from other active mechanisms are often excluded from the physically sensed transformation variable, necessitating several empirical corrections to improve accuracy.

Invoking the Carnot limit—a corollary constraint of the second law—as [32,33]

(9) Energy added=Available energy+Unavailable energy,

a phenomenological transformation metric $w_{phen}$ that indicates the simultaneous contributions of all active processes is the sum

(10) $$w_{phen}=w_{meas}+∆w_{phen}\;$$

of the measurable (or physically sensible) transformation metric $w_{meas}$ and the hitherto unobservable instantaneous contributions ${∆w}_{phen}$ which can be in the form of gradual deviations, fluctuations and instabilities. Here, $w_{phen}$ is the actual transformation corresponding to the energy added/extracted (left hand side term in Equation (9)), $w_{meas}$ is the apparent transformation—usually representative of the primary work interaction(s)—corresponding to the available energy (the first right hand side term in Equation (9)) and ${∆w}_{phen}$ is the unobservable transformation corresponding to the unavailable energy (the second right hand side term in Equation (9)). Given that ${∆w}_{phen}$ cannot be directly measured or sensed via the system/process variables that are observable, the actual (phenomenological) transformation path/trajectory $w_{phen}$ is directly unobservable. In Section 6, we will outline a procedure to evaluate $w_{phen}$ for use in sensing applications.

### 5.3. Phenomenological Transformation–Phenomenological Entropy Generation Theorem

Proposed is a theorem related to the original DEG theorem for application in instantaneous material/system transformation including system formation and decomposition.

**Theorem** **1.** *Given a non-monotonic material transformation consisting of r active processes with process energies $p_{r}$ dependent on the thermodynamic state **Z** and time-varying phenomenological variables $\zeta_{r}=\zeta_{r}(t)$ such that $p_{r}=p_{r}[Z,\;\zeta_{r}\;(t)],r=1,\;2,\;.\;.\;.\;,\;n$, the phenomenological transformation*

(11) $${\dot{w}}_{phen}=\sum_{phen}B_{phen}{\dot{S}}_{phen}=B_{W,r}\dot{S}{’}_{W,r}(p_{r})+B_{\mu T}\dot{S}{’}_{\mu T}\left(p_{\mu T}\right)$$

*is a linear combination of the rate of phenomenological entropy $S_{phen}^{\prime }$ generated by the active processes, where the transformation coefficients $B_{W,r}=\frac{\partial w_{phen}}{\partial {S^{\prime }}_{W,r}};B_{\mu T}=\frac{\partial w_{phen}}{\partial {S^{\prime }}_{\mu T}}$ are slopes of phenomenological transformation $w_{phen}$ with respect to the work (internal, external and flow) entropy rate $\dot{S}{’}_{W,r}=\sum \frac{Yr{\dot{X}}_{r}}{T}$ and MicroStructuroThermal MST entropy rate $\dot{S}{’}_{\mu T}=\frac{-S\dot{T}}{T}$, respectively.*

**Proof.**  Via the proportionality between degradation and entropy generation per the DEG theorem, with entropy generation as the sum of the contributions by the active processes $S\prime =\sum_{i}{S^{\prime }}_{i}$, and recalling Definitions 1–6 (in Section 2) and the considerations that led to the PEG theorem [27] Equation (4), system/material degradation is expressed as

(12) $$\delta w={\delta w}_{phen}-{dw}_{rev}\;\;\mathrm{for}\;{\delta S}^{\prime }={\delta S}_{phen}-{dS}_{rev}$$

the difference between phenomenological transformation (or degradation) and reversible transformation (or degradation). Similar to maintaining ${\delta S}^{\prime }\geq 0$ mandated by the second law, Equation (12) must maintain a monotonic δw to conform with DEG theorem conditions. Substituting the rate forms of Equations (4) and (12) into DEG Equation (1) yields

(13) $$\dot{w}={\dot{w}}_{phen}-{\dot{w}}_{rev}=B_{phen}{\dot{S}}_{phen}-B_{rev}{\dot{S}}_{rev}=B_{W,r}\dot{S}{’}_{W,r}+B_{\mu T}\dot{S}{’}_{\mu T}-B_{rev}{\dot{S}}_{rev}$$

a re-statement of the DEG theorem but in different variables. The transformation coefficients, as defined by Equation (2), $B_{W,r}=\frac{\partial w_{phen}}{\partial {S^{\prime }}_{W,r}};B_{\mu T}=\frac{\partial w_{phen}}{\partial {S^{\prime }}_{\mu T}};B_{rev}=\frac{dw_{rev}}{dS_{rev}}$ pertain to internal/external work (and flow) interactions, internal free energy dissipation MST and reversible change, respectively. These coefficients can be evaluated as the slopes of $w_{phen}$ versus phenomenological entropy generation components ${S^{\prime }}_{phen}$.

From Equation (13), the reversible transformation rate

(14) $${\dot{w}}_{rev}=B_{rev}{\dot{S}}_{rev}$$

directly correlates with the reversible entropy rate. The reversible entropy change ${dS}_{rev}$ as a linear function between two states can be obtained as the quotient of a standardized energy (e.g., standard Gibbs of formation) and temperature. Reversible transformation ${dw}_{rev}$, like reversible entropy, is constant over a continuous process time interval. The reversible changes ${dw}_{rev}$ and ${dS}_{rev}$, lack the instantaneous nonlinear characteristics of active dissipative processes and only serve as theoretical (maximum or minimum transformation/work) limits, necessary for degradation quantification. Particularly, for energy storage systems—such as batteries—in which degradation is often measured via an increase/decrease in energy storage capacity relative to a reference, the reversible terms of Equations (13) and (14) are used [20,21,26].

The phenomenological transformation rate ${\dot{w}}_{phen}$ and entropy generation rate ${\dot{S}}_{phen}$ are instantaneous accounts of all active processes along the observable transformation path of the system, including the contributions of all active mechanisms. **While degradation** w **involves energy dissipation, transformation** $w_{phen}$ **includes energy conversion and dissipation**. From Equation (13), over a time period beginning with zero initial states, the accumulated phenomenological transformation is

(15) $$w_{phen}=\sum_{phen}B_{phen}{S\prime }_{phen}=B_{W,r}S{’}_{W,r}+B_{\mu T}S{’}_{\mu T}$$

where $B_{W,r}S{’}_{W,r}=B_{W,1}S{’}_{W,1}+B_{W,2}S{’}_{W,2}+\ldots$ for multiple concurrent active independent processes. □

**Figure 1:**
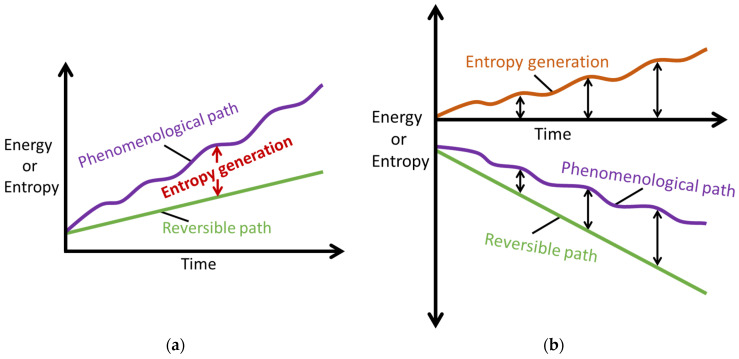
Illustrations of the phenomenological entropy generation theorem for (**a**) energy addition/system formation $0<{dS}_{rev}\leq {\delta S}_{phen}$ and (**b**) energy extraction/system loading ${dS}_{rev}\leq {\delta S}_{phen}<0$. Reproduced from [27].

Phenomenological entropy generation specifies active mechanisms which when combined with a transformation measure, via an interpretation of the DEG theorem, yield the characteristic instantaneous transformation model, Equation (15). During energy addition, system formation or other positive transformation ${\dot{w}}_{phen}>0$, ${\dot{S}\prime }_{phen}>0$ and ${\dot{S}\prime }_{W}>0$ and vice versa for energy extraction, system loading or other negative transformation. $\dot{S}{’}_{\mu T}$ can be negative or positive depending primarily on temperature change. In later sections, we will highlight these effects on the transformation coefficients. Equation (15) is the fundamental *transformation–phenomenological entropy generation* relation, applies to decomposition/degradation and formation/healing processes and is universally instantaneous, according with the second law, the PEG theorem and the DEG theorem. Figure 1 illustrates these concepts.

## 6. Transformation/Degradation Analysis via TPEG Methods

### Generalized Transformation Analysis Procedure

The procedure for the demonstrated structured transformation/degradation analysis methodology [20,21,22,23,24,25,26] is as follows:

(i) Identify a measurable transformation parameter, *w*, that is observable to the transformation characteristics;
(ii) Measure or estimate the transformation $w_{meas}$, and evaluate the concurrent phenomenological entropy generation terms $S_{W,r}^{\prime }$ and ${S\prime }_{\mu T}$ due to the active processes during the interactions;
(iii) Obtain the coefficients $B_{W,r}$ and $B_{\mu T}$ by correlating transformation $w_{meas}$ increments, accumulations or rates to phenomenological entropy generation increments, accumulation or rates (model calibration step);
(iv) Re-combine the now-evaluated (or calibrated) coefficients $B_{W,r}\;and\;B_{\mu T}$ with entropies $S_{W,r}^{\prime }$ and ${S\prime }_{\mu T}$ via Equation (15), to obtain instantaneous transformations in $w_{phen}$ which were hitherto unobservable.

## 7. Transformation and Degradation Analysis Examples

In this section, formulations are adapted to several distinct example system–process interactions, with real-world data. Equations, figures and experimental results are reproduced from references [20,21,22,23,24,25,26] in which the details of each specific system characterization, recommended to the reader, were recently presented. Battery (re)charge will demonstrate positive transformation, and other examples will demonstrate degradation. While each application showed system-specific characteristics, overall trends are as anticipated in the theoretical formulations. In all systems analyzed, a goodness of fit $R^{2}$ ≈ 1 was obtained between model and experimental data in step iii in Section 6.

### 7.1. Friction Sliding of Copper against Steel at Steady Speed—Steady State

Table 2 applies the methods of Section 6 to the wear analysis of a copper rider sliding against a steel countersurface under boundary lubrication. The normal load was 97 N and steady speed $\dot{x}$ = dx/dt = 3.3 ms^−1^ [23]. The steady-state friction force F and temperature *T* were measured during sliding (row 2) to render dT=0; hence, ${S\prime }_{\mu T}=0$, and *frictional* entropy $S_{W}^{\prime }=\int_{t_{o}}^{t_{f}}\frac{F\dot{x}}{T}dt$ (row 3). Via step (iii) of Section 6, the normalized steady-state wear—the degradation measure—was correlated (curve-fitted) with frictional entropy to obtain the DEG coefficient (row 4). A near-linear correlation is evident between measured wear and single-variable entropy generation, per the DEG theorem, Equation (3).

### 7.2. Lubricants—Grease

Grease is a semi-solid lubricant (sometimes called a Bingham solid or liquid) typically used in heavy load applications requiring semi-permanent lubrication. Over time, the loss of shear strength (or stress accumulation) in grease can result in the catastrophic failure of lubricated interfaces. For grease shearing between solid interfaces, the boundary work rate is $Y\dot{X}=V\tau \dot{\gamma }$—appropriately termed the rheological or shear power—in the absence of oxidation (*dN_k_* = 0) [13,34,35]. Table 3 demonstrates the proposed degradation analysis procedure described in Section 6, for lubricant grease shearing. Two different-composition greases—multipurpose lithium grease, NLGI 2, and aircraft lithium grease, NLGI 4—were sheared in a cup using an impeller at constant strain rates. In Table 3, the measured shear stresses and temperatures (row 2) and strain rates and grease material properties for both greases were substituted into the terms in Equation (5) to obtain the shear entropy and MST entropy densities (row 3) which were, in turn, substituted into Equation (7) to obtain the degradation model (row 4). Via step (iii) of Section 6, the DEG coefficients were obtained by simultaneously correlating (i.e., curve fitting) both time-based entropies with concurrently accumulated shear stress, the transformation measure.

The graphical representation of the model presents the instantaneous transformations of both greases in a 3D space, with each grease’s transformation trajectory lying on a different hyperplane. Recalling the dissipation factor, Equation (7), which is the ratio of MST entropy to work entropy—obtainable from the horizontal dimensions of the hyperplanes—the NLGI 2 grease underwent less dissipation, and hence degradation, than the NLGI 4 grease. Via step (iv), the DEG coefficients from step (iii) were re-combined with the phenomenological entropies to yield the phenomenological shear stress (row 5) which shows previously unobservable fluctuations. These fluctuations are direct contributions of the internal temperature-induced microstructural changes (including thermo-elasticity and thermo-plasticity) to the shear stress allowing the observer to monitor the contributions of all the active mechanisms via one transformation measure.

### 7.3. Energy Storage Systems—Li-ion, Ni-MH, Pb-Acid Batteries, Supercapacitors and Fuel Cells

Energy storage systems such as batteries are ubiquitous. Ready availability and safety are two major issues plaguing the energy storage industry. The effects of capacity fade and instability can be dire, necessitating a consistent and accurate analysis approach. For electrochemical system discharge and charge, Helmholtz–Gibbs coupling [27] yields $\mu \dot{N}=vI$ termed the Ohmic power. For rechargeable energy systems that use a degradation measure defined with reference to a known ideal output/capacity, a reversible component obtained using the reversible current as $q_{rev}=\int_{t_{0}}^{t}I_{rev}dt$—typically negligible in other systems—rendered the initial/reference capacity. Reversible current $I_{rev}$ can be obtained as the initial current in a constant-resistive load discharge step, when the battery is at its healthiest. This allowed for the convenient choice of the widely used capacity fade as degradation measure, with charge content as transformation measure [20,26,31].

**Figure 2:**
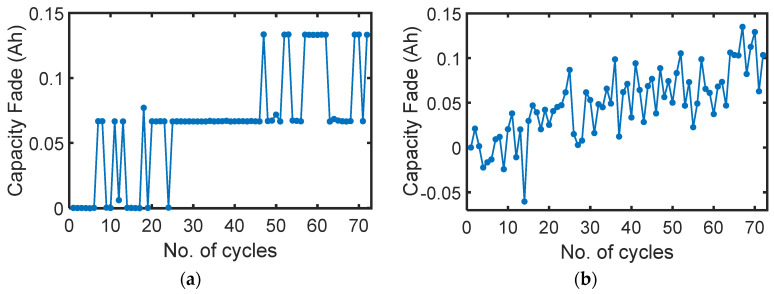
Cyclic capacity fade measured during consistent cycling of Samsung single-cell 3.6 V, 2.5 Ah lithium-ion polymer battery [31]. (**a**) Discharge steps; (**b**) recharge steps.

In a rechargeable battery, the recharge step can reverse the capacity fade of recent cycles. These recharge steps are considered positive transformation, restoring the health of the battery. This is in addition to each recharge step being an energy-addition step without which the battery would remain in a degraded state. Figure 2, from data presented in [31], shows the cyclic capacity fade—the difference between the initial/reference total charge content in the battery and the present total charge content—measured on a Samsung single-cell 3.6 V, 2.5 Ah lithium-ion polymer battery. Figure 2a shows that after the first six discharge steps, discharge capacity dropped by 0.06 Ah; then, the 9th and 10th cycles recovered this loss. Similar behavior is observed in subsequent discharge steps and in the recharge steps (Figure 2b). Note the difference in the trajectories of the discharge capacity fade and recharge capacity fade but the similarity in their initial and final values. Both steps, energy extraction and energy addition, are fundamentally different and impact the battery’s health differently. Note that degradation can and often occurs during energy addition, especially at high rates as commonly experienced in the fast charging of batteries.

Table 4 applies the new TPEG methodology (the methods of Section 6) to the *inconsistent* cycling of a single-cell 3.7 V 11.5 Ah lithium-ion polymer battery. One randomly chosen cycle consisting of a discharge step, followed by a charge step, is shown. Each non-header row in Table 4 represents a transformation analysis step. In step iii, the transformation measure is the instantaneous charge content in the battery which increases during charge/recharge and decreases during discharge. The measured charge content is obtained via Coulomb counting as $q=\int_{t_{0}}^{t}Idt$, where *I* is instantaneous discharge or (re)charge current. Capacity fade (the last row), the degradation measure, is obtained as the difference between phenomenological charge content and reversible charge content according to Equation (13).

In the graphical representation of the transformation model, both discharge and charge trajectories lie in the same 3D space but on different hyperplanes. Even though the charge accumulation is shorter than the discharge accumulation, it is evident that the lower-current charge step is less dissipative than the higher-current discharge step via a lower dissipation factor *J*. With an estimated reversible entropy, entropic efficiency $\eta_{S\prime }$, a measure of Coulombic efficiency, also favors the charge step. Observed behaviors accord with theoretical anticipations presented previously. Similar results were obtained and published for seven lithium-ion batteries [20,22,31], four heavy-duty lead–acid batteries [22,26], a nickel–metalhydride battery, a supercapacitor and a fuel cell [21]. See Section 9.2 for a battery thermal runaway characterization.

### 7.4. General Fatigue—Cyclic Bending and Torsion of Metal Rods

Similar to the grease application discussed in Section 7.2, generalized stress- and strain-based PEG and DEG models were obtained [24] for the dynamic loading of solid components and demonstrated using the experimental low-cycle bending and torsional fatigue of stainless steel SS 304 rods [8]. Here, work δW=YdX=Vσ:dε (also termed “load” in accordance with fatigue loading), where σ is the stress tensor, and ε is the elemental strain, with elastic and plastic components: $\sigma =\sigma_{e}+\sigma_{p}$, $\varepsilon =\varepsilon_{e}+\varepsilon_{p}$. Strain is chosen as the performance indicator [24]. Table 5 applies the TPEG methods to metal fatigue. Each row in Table 5 represents a TPEG analysis step. In step iii, the transformation measure is strain accumulation $\varepsilon =\int_{t_{0}}^{t}\dot{\varepsilon }dt$ obtained via the time integration of strain rate $\dot{\varepsilon }$. In the graphical representation of the transformation model, both bending and torsion transformation trajectories lie in the same 3D space but on different hyperplanes. The less dissipative bending load, with the narrower hyperplane, has a lower dissipation factor *J*.

In the last row, the internal structural mechanisms, such as crack propagation, that led to the sudden rise in temperature are not observable in the measured cumulative strain (blue plot). Via the re-combination of the transformation coefficients with the entropies, phenomenological cumulative strain (purple plot) bears the fluctuations that eventually lead to now-observable failure onset. As with frictional wear, grease degradation and battery aging, observed behaviors are anticipated by the models and consistent interpretations of methodology features.

### 7.5. Pump Flow—Pressure and Flow Rate (Internal Energy)

Measured on a three-phase 11 kW 16 l/s centrifugal motor pump operating at steady state are inlet/exit pump pressures and the flow rate [36], which are plotted versus time in the second row of Table 6. Pump power $Y\dot{X}=M_{T}\omega$, where $M_{T}$ is torque, and ω is rotational speed. The analysis methods of Section 6 applied to pump transformation and degradation are outlined in Table 6, with each step of the proposed analysis method in each non-header row. The transformation measure in step iii is pressure drop, the difference between exit pressure and inlet pressure. Similar to prior examples, the pump’s transformation trajectory lies on a hyperplane in the 3D domain.

## 8. Elements of the TPEG Methodology

### 8.1. PEG Terms: Work (Including Flow and Reaction) Entropy and MST/ECT Entropy

The third rows (step (ii)) of Table 2, Table 3, Table 4, Table 5 and Table 6 present phenomenological entropy generation (PEG) constituent terms for the various systems demonstrated in this study. The work entropy (single-variable entropy generation) ${S^{\prime }}_{W,r}$—shear entropy in greases, Ohmic entropy in electrochemical systems, load entropy in fatigue loading, flow and work entropies in loaded open systems—is monotonically curvilinear, confirming prior entropy-based models that employ this term only [8,9,13,22,34], whereas the MST/ECT entropy (red plots) shows instantaneous transients and nonlinearities that characterize the internal fluctuations in the systems that were hitherto unobservable. Noting the order of magnitude difference between MST/ECT and work entropies, the work entropies are significantly higher. As indicated by the free energy transformation, while the work entropy is the minimum entropy generation required for the process to occur, the MST/ECT entropy generally has an adverse impact in non-thermal systems, intensifying degradation. In addition, the sudden rise in the magnitude of the MST entropy ${S^{\prime }}_{\mu T}$ just before fatigue failure (row 3 of Table 5) is not evident in the work entropy ${S^{\prime }}_{W,r}$. Instability and critical phenomena are discussed in Section 9.

### 8.2. Degradation, A Geometric Problem: TPEG Trajectories, Hypersurfaces, and Domains

This article presented a multi-dimensional space that linearly characterizes a real system’s nonlinear phenomenological transformation. Rows 4 (step (iii)) of Table 2, Table 3, Table 4, Table 5 and Table 6 show the same three-dimensional representation of vastly different systems (grease, batteries, stainless steel rod and water pump), with identical features. In all five figures—and others excluded here for brevity—the measured data points that define the component’s path during transformation—its **transformation–phenomenological entropy generation (TPEG) trajectory**—lie on tilted **TPEG hypersurface(s).** The orthogonal multi-dimensional space occupied by TPEG trajectories and surfaces is the component’s material-dependent **TPEG domain/space**. Processes with one significant primary interaction, e.g., the unsteady (anisothermal) mechanical shearing of grease, have a three-dimensional TPEG space. Processes with two significant interactions, e.g., the unsteady mechanical shearing of oxidizing grease, have a four-dimensional TPEG domain. Transformation mechanisms with more active processes will require yet higher dimensional TPEG spaces, which are possible mathematically. TPEG trajectories characterize loading conditions (battery discharge/charge rate, grease shear/oxidation rate, metal torsion/bending stress/strain amplitudes, flow rates, etc.). TPEG hypersurfaces characterize system/material composition and dissipative process rates. The TPEG domain defines the operating/aging/failure region, fully specifying the component’s life, transformation and degradation path for all loading conditions and active mechanisms. Proper formulation of the phenomenological entropy generation of the active processes is required to accurately determine contributions to overall entropy accumulation and degradation during system transformation, loading or operation.

#### 8.2.1. TPEG Coefficients

The orientations of the TPEG hypersurfaces yield the TPEG coefficients. Unlike half-theoretical, half-empirical methods which predict the suitability of a system for application using extensive experimental data from several failed samples (under controlled loads), TPEG coefficients can be obtained from one or two representative samples (or first few cycles, for rechargeable energy systems) and applied to all other systems of the same material(s) undergoing similar in situ processes (or subsequent cycles, for rechargeable energy systems). These coefficients show the system’s true response to active interactions and conditions by quantifying the processes’ dissipative contributions towards degradation and failure.

Internal and external work coefficients $B_{W,r}$ are negative for a positive transformation of the performance indicator: work entropies $S{’}_{W,r}$ are negative during the loading and loss/depletion of active species. For grease, $B_{W,r}$ is negative given that shear stress is always positive; for batteries, $B_{W,r}$ is positive given that charge transfer is negative during discharge and positive during recharge. MST coefficient $B_{\mu T}$ has a varying sign characteristic. To understand $B_{\mu T}$ sign changes, rearrange Equation (15) into the form

(16) $$B_{\mu T}=\frac{1}{{S\prime }_{\mu T}}\left(w_{phen}-B_{W,r}{S^{\prime }}_{W,r}\right)$$

Note that the phenomenological degradation measure $w_{phen}$ (e.g., $\varepsilon_{phen}$, last row of Table 5) fluctuates about the work-based measure $B_{W,r}{S\prime }_{W,r}$, making the parenthesis expression in Equation (16) fluctuate about zero during operation. This verifies that the MST entropy ${S^{\prime }}_{\mu T}$ includes instantaneously transient phenomena [23] such as thermo-elasticity and self-reorganization, among others. Thus, ${S\prime }_{\mu T}$, being a measure of internal fluctuations in the system, can be positive or negative.

#### 8.2.2. Entropy Generation Subspace and Reversible Transformation Subspace: Dissipation Factor *J* and Entropic Efficiency $\eta_{S\prime }$

**Figure 3:**
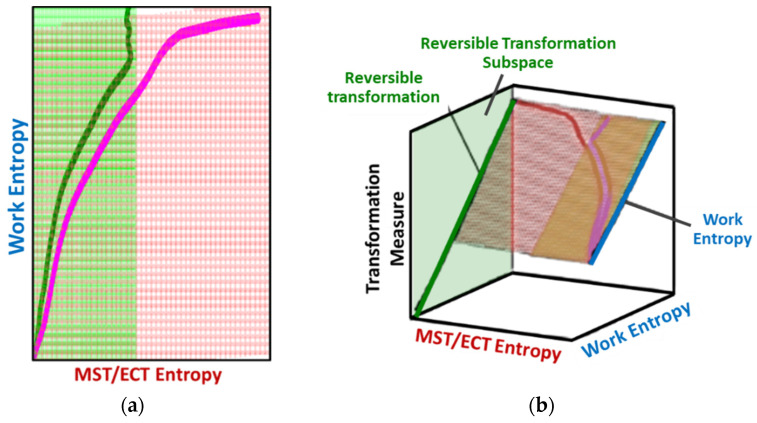
TPEG domain/space and subspace representations of the (**a**) $dissipation\;factor=\frac{MST/ECT\;entropy}{Work\;entropy}=\frac{Depth}{Length}$ in the entropy generation subspace EGS and (**b**) $entropic\;efficiency=\frac{Work\;entropy}{Reversible\;entropy}=\frac{Length}{Ideal\;length}$ in the reversible transformation subspace. The green and pink curves are the transformation trajectories. Axes are not to scale.

Via the simultaneous transition of the system along the MST ${S\prime }_{\mu T}$ and work entropy ${S\prime }_{W}$ axes of the TPEG space, all systems studied showed a useful relationship between ${S\prime }_{\mu T}$ and ${S\prime }_{W}$. This is visualized as a projection of the tilted hyperplane and trajectories onto the entropy generation ${S\prime }_{\mu T}$—${S\prime }_{W}$ subspace [37], as shown in Figure 3a. A component having a TPEG domain with large transformation measure dimension (the w direction or height) and small MST/ECT entropy dimension (the depth), relative to work entropy dimension (the length or width) will do more useful work before failure. The TPEG space and Equation (7) indicate that a low dissipation factor *J* (narrow-surfaced domain) is always desirable for slow degradation; see Figure 3a.

Recall the definition of entropic efficiency as the ratio of actual work/load entropy to reversible (ideal) entropy. In Figure 3b, with an ideal system having no MST/ECT entropy, the actual trajectory spanning both horizontal axes (the curvilinear lines on the planes) is translated/flattened into a virtual (and theoretical) reversible transformation trajectory (green line in Figure 3b) on the reversible transformation subspace. The reversible transformation line is a straight line traversing the two-dimensional transformation measure work entropy ($w{—S\prime }_{W}$) pair of axes only and of the same overall length as or longer than the phenomenological trajectory. Hence, the energy available for work (represented by the blue line in Figure 3b) depends on the fraction of the theoretical total (the green line) lost to internal microstructural and thermal transformations and entropy generation. As anticipated by formulations in references [27,31] and Section 4.2, high entropic efficiency $\eta_{S\prime }$ is always preferred for slow degradation and optimum performance.

## 9. Instability and Critical Phenomena

### 9.1. MST/ECT Entropy and Critical Failure Entropy ${S\prime }_{CF}$

**Figure 4:**
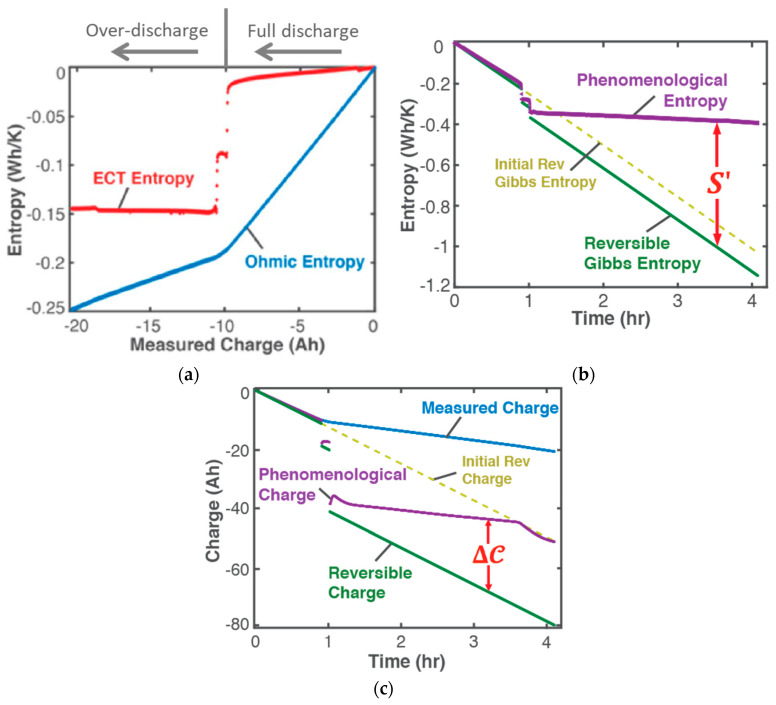
(**a**) Ohmic and ECT entropies during the over-discharge of a lead–acid battery. The sudden drops in ECT entropy coincided with a sudden 2-step transition to over-discharge, following full discharge. (**b**) Phenomenological entropy (the sum of Ohmic and ECT entropies) and reversible entropy showing the 2-step drops violating the second law momentarily when $S_{rev}>S_{phen}$. (**c**) Phenomenological charge and reversible charge showing the 2-step drops violating the DEG theorem momentarily when $w_{rev}>w_{phen}$. Reproduced from [26].

A corollary of the DEG theorem asserts that “if a critical value of degradation measure exists at which failure occurs, there must also exist critical values of accumulated irreversible entropies” [28]. Naderi, Amiri and Khonsari [8,9,10,11], via experimental data, showed the existence of a material-dependent fatigue fracture entropy *FFE* evaluated as accumulated load entropy at failure onset (using constant plastic strain amplitude). References [8,9,24] verified similar magnitudes of cumulative load entropy ${S\prime }_{W}$ for both the bending and torsion of the SS 304 steel specimen. Commonly used physical fatigue tools like stress–life σ—N and strain–life ε—N curves, with constant or variable stress and strain amplitudes, do not exhibit the critical phenomenon. The TPEG domain, in contradistinction, shows a distinct and consistent location of the critical onset of failure, a phenomenon also observed at the sudden transition to over-discharge in lead–acid batteries [26] (Figure 4). TPEG’s critical failure entropy is inherent in the second law as given in Equation (4). The entropy generation evolution criterion for stable spontaneous process continuity $S\prime =S_{phen}-S_{rev}\geq 0$ where $S_{rev}\leq S_{phen}$ is the stability criterion. Then, an abrupt downward spike in $S_{phen}$ due to sudden instability results in the second law-prohibited negative entropy generation, indicating a discontinuity in the process. This is observed in row 3 (step (ii)) of Table 5, where the abrupt increases in MST entropy storage magnitudes just before failures coincide with the sudden rises in specimen temperatures. At the point of catastrophe, micro-cracks join into a macro-crack which extends in an unstable manner. The newly created free surface from the crack would alter heat flows and thus temperature. Via the *B* coefficients, these abrupt changes are transferred to phenomenological strains, the last row (step (iv)) of Table 5, introducing the critical feature to the otherwise steady degradation measures (normal and torsional strains).

This feature is also observed in the sudden drop in ECT entropy at the transition from full discharge to over-discharge of electrochemical energy systems, e.g., lead–acid batteries [22,26] (see Figure 4a). Hence, MST/ECT entropy measures the system’s instantaneous instabilities and ultimate failure. In other forms of loading including the thermal and chemical cycling of components, the significance of MST/ECT entropy is further underscored by the limited safe operating temperature ranges specified by device manufacturers to prevent runaway events. In Figure 4b, the phenomenological entropy (purple plot) is the sum of the Ohmic and ECT entropies in Figure 4a. Prior to the first drop, the reversible entropy (yellow broken lines plot) is less than the phenomenological entropy ($S_{rev}\leq S_{phen}<0$) as required by the second law for stability. The sudden drops at the transition to over-discharge caused reversible entropy to exceed phenomenological entropy $(S_{rev}>S_{phen}$), rendering a negative entropy generation $S^{\prime }=S_{phen}-S_{rev}<0$. The 6 V lead–acid battery became unstable momentarily but quickly stabilized to a new low voltage of 1.7 V, its new operational voltage which defined a new reversible entropy (the green plot) to allow for continuous operation for three more hours in the over-discharge phase.

Inheriting the entropy generation stability criterion via the DEG theorem, a transformation/degradation stability criterion is given by $w=w_{phen}-w_{rev}\geq 0$ where $w_{rev}\leq w_{phen}<0$. Shown in Figure 4c are sudden drops in phenomenological charge (purple plot) during the transition to over-discharge, marking instabilities.

### 9.2. Thermal Runaway in Batteries

**Figure 5:**
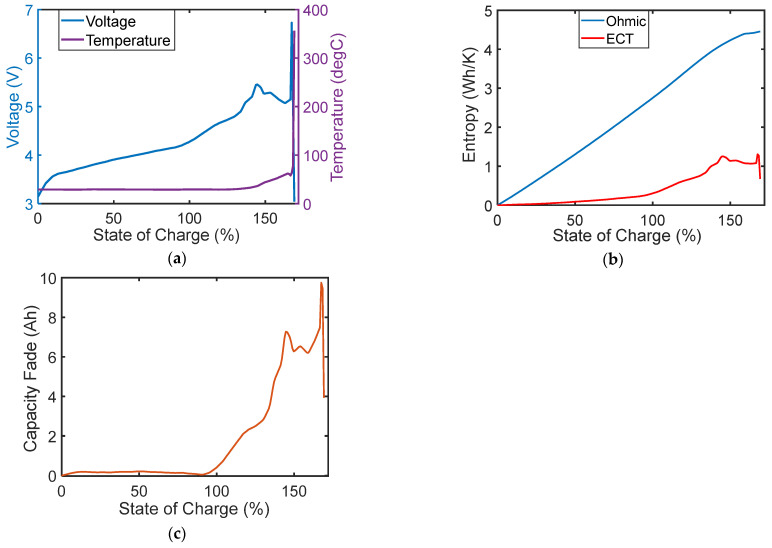
TPEG analysis/monitoring of overcharge-induced thermal runaway in a 20 Ah commercial lithium-ion battery, as a function of the state of charge SOC. (**a**) Voltage and temperature data extracted from [39]. (**b**) Phenomenological entropy components: Ohmic and ECT. (**c**) Instantaneous capacity fade.

Feng et al. [38] presented a detailed review of thermal runaway in electric vehicle (EV) lithium-ion batteries. The authors discussed the critical safety issues in EV applications accompanying the increasing energy densities of commercial Li-ion batteries, characterizing the reaction kinetics via energy release. Ouyang et al. [39] investigated the overcharge-induced thermal runaway of a 20 Ah commercial lithium-ion battery, highlighting four phases of capacity fade. Figure 5a reproduces the voltage and temperature data from reference [39]. (The dataset was extracted using Rohatgi’s Webplotdigitizer [40]). Ouyang et al. observed that the battery’s temperature was relatively steady until the state of charge SOC exceeded 120%, followed by battery swelling at 145% SOC, rupture at 167% and thermal runaway at 169%. They also showed that the battery’s internal resistance was steady until the sudden rise after 120% SOC.

Employing the TPEG methodology—Figure 5b,c—instantaneous capacity fade is evaluated, showing an abrupt rise just before 100% SOC which continues and eventually leads to the failure events. The TPEG capacity fade (Figure 5c) combines the voltage and temperature responses (Figure 5a) via the Ohmic and ECT entropies (Figure 5b) and the TPEG coefficients to monitor the true transformation of the battery, signaling the onset of instability well before the battery’s internal resistance, temperature and physical deformation indications. Note in Figure 5b that the Ohmic entropy shows a minimal instability characteristic, whereas the ECT entropy incorporates the instantaneous effects of both voltage and temperature changes. Figure 5c shows the capacity fade plotted versus the SOC. See Section 7.3 and previous TPEG battery applications in the references list for details.

## 10. Discussion

A combination of the DEG and PEG theorems provides a structured approach to system/component transformation/aging/degradation/failure modeling, removing the need for several, often expensive experimental measurements which require curve fits, statistical corrections and multiple analysis tools. The new TPEG methodology presented herein has accurately and consistently described a component’s transformation levels during operation or manufacture. The method converts non-monotonic transformation assessment into a geometry problem, using a convenient multi-dimensional physics-based *measure* versus *phenomenological entropy generation* space, since entropy-generating processes underlie the active transformations. For optimization, the MST entropy should be minimized. Bejan et al. [4,5,41] propose thermal optimization strategies.

## 11. Summary and Conclusions

“Transformation and Degradation Thermodynamics” was formulated from an ab initio combination of the phenomenological entropy generation (PEG) theorem—derived from fundamental irreversible thermodynamics—with the degradation–entropy generation (DEG) theorem, for universal system transformation analysis. Active unsteady transformations were instantaneously resolved, and a transformation–phenomenological entropy generation (TPEG) theorem was proposed, highlighting the significance of the *MicroStructuroThermal MST* entropy (*ElectroChemicoThermal ECT* entropy for electrochemical power systems) for degradation and transformation. Diverse classes of uncontrolled system–process interactions—frictional wear, grease degradation, battery aging, metal fatigue and pump operation—showed the TPEG-predicted linearity between transformation measures and phenomenological entropy generation constituents, with a statistical fit R^2^ ≈ 1 indicating near-100% accuracy (near-perfect correlation between theory and uncontrolled experiments). Application to system instability and critical phenomena was presented. The flexibility of degradation/transformation parameter selection was demonstrated. New parametric and geometric features—dissipation factor and entropic efficiency—were discussed. The TPEG (DEG + PEG) methodology can directly compare designs and materials for manufacturing and loading applications, in addition to in situ diagnostic performance/health monitoring and optimization. This article successfully verified theory with non-intrusive measurements of temperature and active process parameters, with consistent results. Without being system- or material-dependent, the TPEG methodology is universal, system- and material-characteristic, consistent and readily adaptable to all systems undergoing real-world dynamic loading, energy addition and/or system formation.

## Figures and Tables

**Table 1:** Single-variable entropy generation $\delta {S\prime }_{W,r}$ for various systems.

Work	$\delta {S\prime }_{W,r}$
Frictional	$\frac{F_{f}dx}{T}$
Magnetic	$\frac{BdM}{T}$
Shear	$\frac{V\tau d\gamma }{T}$
Electrical	$\frac{vdq}{T}$
Rotational shaft	$\frac{M_{T}\omega }{T}$
Chemical	$\frac{\sum \mu_{k}dN_{k}}{T}$
Flow	$\frac{\sum \left[{\left({hdN}_{e}\right)}_{exit}-{\left({hdN}_{e}\right)}_{inlet}\right]}{T}$

**Table 2:** Pseudo-steady-state frictional wear analysis via the degradation–entropy generation methodology.

#	Characterization Step	Model and Graphical Representation
(i)	Measured or input data	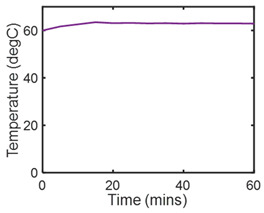
(ii)	**Phenomenological Entropy Generation** Frictional entropy ${S^{\prime }}_{W}=-\int_{t_{0}}^{t}\frac{F\dot{x}}{T}dt$ MST entropy ${S^{\prime }}_{\mu T}=0$	${S\prime }_{phen}={S^{\prime }}_{W}$ 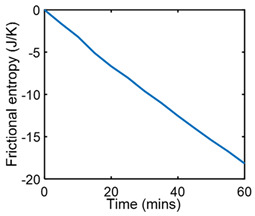
(iii)	Degradation–Entropy Generation Material wear is the degradation measure. Slope yielded $B_{W}=-0.0366$ K/J	**Degradation model: ** $w=B_{W}{S^{\prime }}_{W}$ 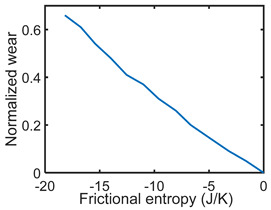

**Table 3:** Grease analysis via the transformation–phenomenological entropy generation methodology. ρ is grease density, *c_γ_* is specific heat capacity at constant shear, *τ* is shear stress, *γ* is shear strain, $\beta ={\left(\frac{\partial \tau }{\partial T}\right)}_{\gamma ,N}=\alpha G\prime$ is the thermal stress coefficient, where $\alpha ={\left(\frac{\partial \gamma }{\partial T}\right)}_{\tau ,N}$ is the thermal strain coefficient, and $G\prime ={\left(\frac{\partial \tau }{\partial \gamma }\right)}_{T,N}$ is the storage modulus [23,27]. *MST: MicroStructuroThermal.* Excluding row 4 (step (iii)) where both greases are overlaid on the same set of axes, the plots on the left are for NLGI 4 grease and on the right are for NLGI 2 grease. Reproduced from [23].

#	Characterization Step	Model and Graphical Representation
(i)	Measured or input data	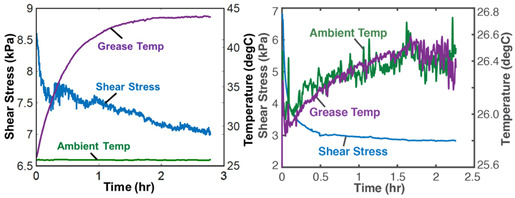
(ii)	**Phenomenological Entropy Generation** MST entropy density ${S^{\prime }}_{\mu T}=-\int_{t_{0}}^{t}\left(\rho c_{\gamma }lnT+\beta \gamma \right)\frac{\dot{T}}{T}dt$ Shear entropy density ${S^{\prime }}_{W}=-\int_{t_{0}}^{t}\frac{\tau \dot{\gamma }}{T}dt$	${S\prime }_{phen}={S^{\prime }}_{\mu T}+{S^{\prime }}_{W}$ 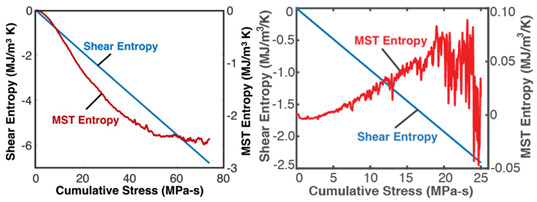
(iii)	**Transformation–Phenomenological Entropy Generation** Shear stress accumulation is the trans-formation measure. Orthogonal slopes yielded $B_{W}=-10.4$$\;\mathrm{Pa}-s\;K/J\;\mathrm{and}\;B_{\mu T}=-0.03$$\;\mathrm{Pa}-s\;K/J\;\mathrm{for}\;\mathrm{NLGI}\;2\;\mathrm{grease}\;\mathrm{and}\;B_{W}=-10.4$$\;\mathrm{Pa}-s\;K/J\;\mathrm{and}\;B_{\mu T}=-0.50$ Pa-s K/J for NLGI 4 grease.	**Transformation model: ** $\int_{t_{0}}^{t}\tau dt=B_{\mu T}{S^{\prime }}_{\mu T}+B_{W}{S^{\prime }}_{W}$ 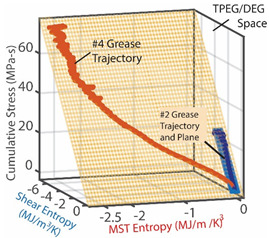
(iv)	Change in shear strength/stress is the degradation measure. $J_{NLGI2}=0.021$ $J_{NLGI4}=$ 0.396	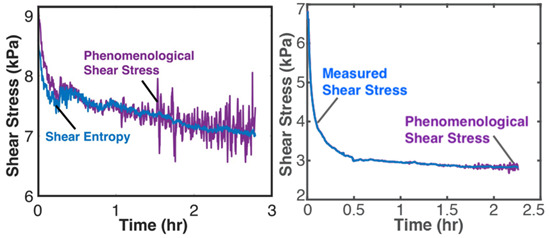

**Table 4:** Battery degradation analysis via the transformation–phenomenological entropy generation methodology. v is battery voltage, *T* is temperature, q is charge content, *I* is current and Δq is capacity fade. *vT*, *ECT: ElectroChemicoThermal*. The arrows indicate process direction, i.e., charge C follows discharge D. Reproduced from [20].

#	Characterization Step	Model and Graphical Representations
(i)	Measured or input data.	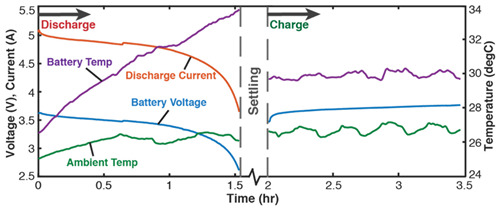
(ii)	**Phenomenological Entropy Generation** *ECT* entropy $S{’}_{vT}=\int \frac{qv}{T}dt$ Ohmic entropy $S{’}_{\Omega }=\int \frac{vI}{T}dt$	${S\prime }_{phen}={S^{\prime }}_{vT}+S{’}_{\Omega }$ 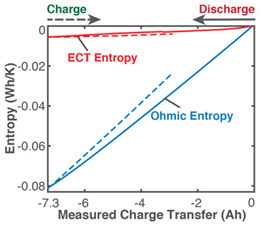
(iii)	**Transformation–Phenomenological Entropy Generation** Charge content is the transformation measure. TPEG coefficients: $B_{\Omega }=76.6$ Ah K/Wh and $B_{VT}=113$ Ah K/Wh for discharge. For charge, $B_{\Omega }=75.5$ Ah K/Wh and $B_{VT}=28.3$ Ah K/Wh.	**Transformation model: ** $q=B_{vT}S{’}_{vT}+B_{\Omega }S{’}_{\Omega }$ 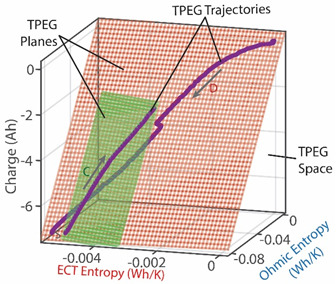
(iv)	Charge capacity fade is the degradation measure. For this cycle, $\Delta q_{disch}=1.3$ Ah $\Delta q_{ch}=0.3$ Ah $J_{disch}=0.063$ $J_{ch}=$0.033 $\eta_{S\prime disch}=$0.72 $\eta_{S\prime ch}=$ 0.93	**Degradation model: **$\Delta q=B_{vT}S{’}_{vT}+B_{\Omega }S{’}_{\Omega }-q_{rev}$ 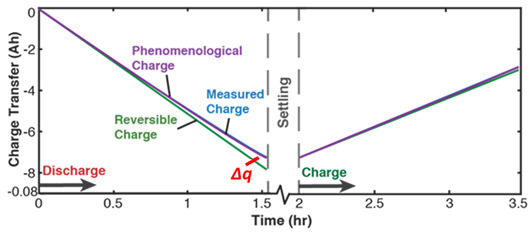

**Table 5:** A summary of the transformation–phenomenological entropy generation methodology for bending and torsion fatigue. ρ is density, $c_{\varepsilon }$ is specific heat capacity, σ is normal stress and ε is normal strain for bending. $\beta ={\left(\frac{\partial \sigma }{\partial T}\right)}_{\varepsilon ,N}=\frac{\alpha }{\kappa_{T}}$ is the thermal stress coefficient, where $\alpha ={\left(\frac{\partial \varepsilon }{\partial T}\right)}_{\sigma ,N}$ is the thermal strain coefficient, and $\kappa_{T}=-{\left(\frac{\partial \varepsilon }{\partial \sigma }\right)}_{T,N}$ is isothermal loadability; ${S^{\prime }}_{\mu T}$ is MST (*MicroStructuroThermal*) entropy, and ${S^{\prime }}_{W}$ is work/load entropy. For torsion, shear stress τ and shear strain γ are used. Excluding row 4 (step (iii)) where both load types are overlaid, plots on the left are for bending, and plots on the right are for torsion. Reproduced from [24].

#	Characterization Step	Model and Graphical Representation
(i)	Measured or input data	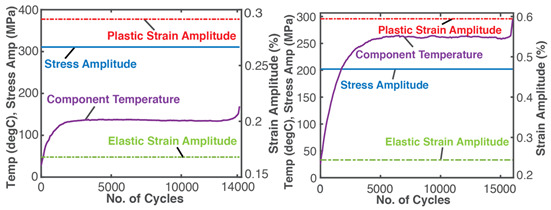
(ii)	**Phenomenological Entropy Generation** ${S^{\prime }}_{\mu T}=-\int_{t_{0}}^{t}\left(\rho c_{\varepsilon }lnT+\beta \varepsilon \right)\frac{\dot{T}}{T}dt$ ${S^{\prime }}_{W}=-\int_{t_{0}}^{t}\frac{N_{dt}\sigma_{N}}{T}:\left[{\dot{\varepsilon }}_{eN}+\left(\frac{1-n\prime }{1+n\prime }\right){\dot{\varepsilon }}_{pN}\right]dt$	${S\prime }_{phen}={S^{\prime }}_{\mu T}+{S^{\prime }}_{W}$ 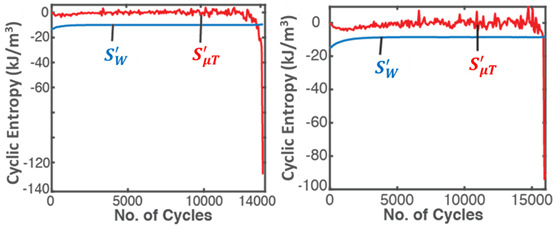
(iii)	**Transformation–Phenomenological Entropy Generation** Strain is the transformation measure. Orthogonal slopes yielded $B_{W}=-0.92$ %m^3^K/MJ and $B_{\mu T}=0.22$ %m^3^K/MJ (bending), and $B_{W}=-1.96$ %m^3^K/MJ and $B_{\mu T}=0.42$ %m^3^K/MJ (torsion), prior to failure onset.	**Transformation model: ** $\varepsilon =\int_{t_{0}}^{t}\dot{\varepsilon }dt=B_{\mu T}{S^{\prime }}_{\mu T}+B_{W}{S^{\prime }}_{W}$ 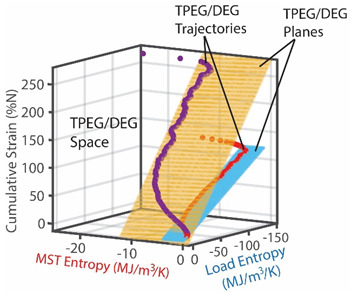
(iv)	Change in strain is the degradation measure. At onset of failure, $J_{bending}\geq 0.04$ $J_{torsion}\geq \;$0.10	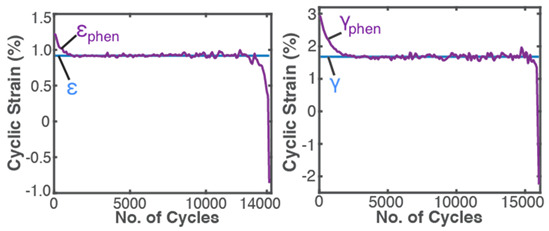

**Table 6:** Characterizing a water pump using the transformation–phenomenological entropy generation methodology.

#	Characterization Step	Model and Graphical Representation
(i)	Measured or input data	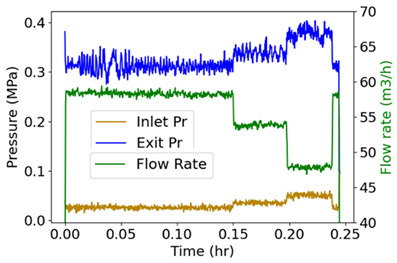
(ii)	**Phenomenological Entropy Generation** Flow entropy ${S^{\prime }}_{N}=\int_{t_{o}}^{t_{f}}\frac{\sum_{\;}^{\;}\left[{\left(\dot{m}h\right)}_{exit}-{\left(\dot{m}h\right)}_{inlet}\right]}{T}dt$ Work entropy ${S^{\prime }}_{W}=\int_{t_{o}}^{t_{f}}\frac{M_{T}\omega }{T}dt$	${S\prime }_{phen}=S_{N}^{\prime }+{S^{\prime }}_{W}$ 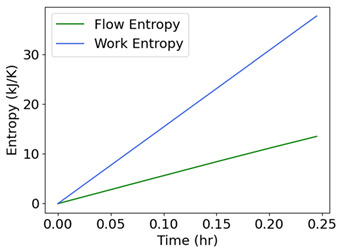
(iii)	**Transformation–Phenomenological Entropy Generation** Pressure drop is the transformation measure. Orthogonal slopes give $B_{N}=-0.057\;$ MPa-h K/kJ and $B_{W}=0.018$ MPa-h K/kJ	**Transformation model: ** $∆P=B_{N}{S^{\prime }}_{N}+B_{W}{S^{\prime }}_{W}$ 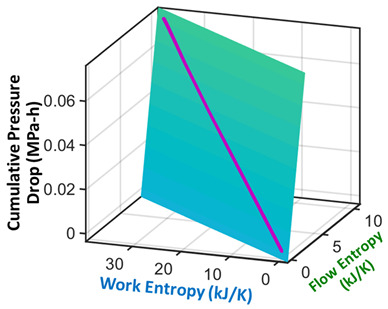
(iv)	Change in pressure drop measures degradation.	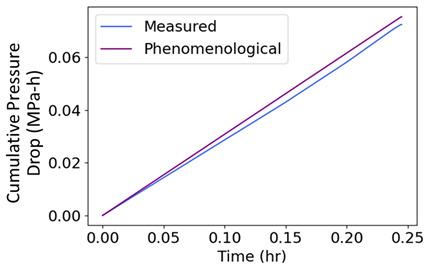

## Data Availability

Data are contained within the article.

## Abbreviations

**Nomenclature**	**Name**	**Unit**
*A*	chemical affinity	J/mol
*B*	DEG coefficient	Ah K/Wh
*C*	charge, charge transfer or capacity	Ah
ΔC	charge fade or capacity fade	Ah
*F*	Faraday’s constant	C/mol
*G*	Gibbs energy	Wh
*I*	discharge/charge current or rate	A
*k_B_*	Boltzmann constant	J/K
*m*	mass	kg
*N*	cycle number	
*N, N_k_*	number of moles of substance	mol
*p*	dissipative process energy	J
*P*	pressure	Pa
*q*	charge	Ah
*Q*	heat	J
*R*	gas constant	J/mol·K
*S*	entropy or entropy content	Wh/K
*S’*	entropy generation or production	Wh/K
*t*	time	s, min, h
*T*	temperature	degC or K
*v*	voltage	V
*V*	volume	m^3^
*w*	degradation measure	
*W*	work	J
**Symbols**		
*μ*	chemical potential	
*ζ*	phenomenological variable	
**Subscripts and acronyms**		
*Ω*	Ohmic	
*0*	initial	
*c, ch*	charge	
*d, disch*	discharge	
*f*	final	
*vT, ECT*	ElectroChemicoThermal	
*μT, MST*	MicroStructuroThermal	
*rev*	reversible	
*irr*	irreversible	
*phen*	phenomenological	
*DEG*	degradation–entropy generation	
*PEG*	phenomenological entropy generation	
*NLGI*	National Lubricating Grease Institute	

## References

[1] Osara J.A. (2019). Thermodynamics of Manufacturing Processes—The Workpiece and the Machinery. Inventions.

[2] Bryant M.D. (2014). Chapter 3: Thermodynamics of Ageing and Degradation in Engineering Devices and Machines. The Physics of Degradation in Engineered Materials and Devices: Fundamentals and Principles.

[3] Lemaitre J., Chaboche J.L. (1994). Mechanics of Solid Materials.

[4] Bejan A., Tsatsaronis G., Moran M.J. (1996). Thermal Design and Optimization.

[5] Bejan A. (2013). Entropy Generation Minimization: The Method of Thermodynamic Optimization of Finite-Size Systems and Finite-Time Processes.

[6] Kuhn E. (1991). An Energy Interpretation of Thixotropic Effects. Wear.

[7] Kuhn E. (1995). Description of the Energy Level of Tribologically Stressed Greases. Wear.

[8] Naderi M., Khonsari M.M. (2010). An Experimental Approach to Low-Cycle Fatigue Damage Based on Thermodynamic Entropy. Int. J. Solids Struct..

[9] Amiri M., Naderi M., Khonsari M.M. (2011). An Experimental Approach to Evaluate the Critical Damage. Int. J. Damage Mech..

[10] Naderi M., Khonsari M.M. (2012). A Comprehensive Fatigue Failure Criterion Based on Thermodynamic Approach. J. Compos. Mater..

[11] Naderi M., Khonsari M.M. (2012). Thermodynamic Analysis of Fatigue Failure in a Composite Laminate. Mech. Mater..

[12] Rezasoltani A., Khonsari M.M. (2014). On the Correlation between Mechanical Degradation of Lubricating Grease and Entropy. Tribol. Lett..

[13] Rezasoltani A., Khonsari M.M. (2016). An Engineering Model to Estimate Consistency Reduction of Lubricating Grease Subjected to Mechanical Degradation under Shear. Tribol. Int..

[14] Aghdam A.B., Khonsari M.M. (2014). Prediction of Wear in Grease-Lubricated Oscillatory Journal Bearings via Energy-Based Approach. Wear.

[15] Sosnovskiy L., Sherbakov S. (2009). Surprises of Tribo-Fatigue.

[16] Sosnovskiy L.A., Sherbakov S.S. (2016). Mechanothermodynamic Entropy and Analysis of Damage State of Complex Systems. Entropy.

[17] Basaran C., Nie S. (2004). An Irreversible Thermodynamics Theory for Damage Mechanics of Solids. Int. J. Damage Mech..

[18] Gomez J., Basaran C. (2005). A Thermodynamics Based Damage Mechanics Constitutive Model for Low Cycle Fatigue Analysis of Microelectronics Solder Joints Incorporating Size Effects. Int. J. Solids Struct..

[19] Basaran C. (2023). Introduction to Unified Mechanics Theory with Applications.

[20] Osara J., Bryant M. (2019). A Thermodynamic Model for Lithium-Ion Battery Degradation: Application of the Degradation-Entropy Generation Theorem. Inventions.

[21] Osara J.A., Bryant M.D. (2021). Performance and Degradation Characterization of Electrochemical Power Sources Using Thermodynamics. Electrochim. Acta.

[22] Osara J.A. (2017). Thermodynamics of Degradation.

[23] Osara J.A., Bryant M.D. (2019). Thermodynamics of Grease Degradation. Tribol. Int..

[24] Osara J.A., Bryant M.D. (2019). Thermodynamics of Fatigue: Degradation-Entropy Generation Methodology for System and Process Characterization and Failure Analysis. Entropy.

[25] Osara J.A., Bryant M.D. (2020). A Temperature-Only System Degradation Analysis Based on Thermal Entropy and the Degradation-Entropy Generation Methodology. Int. J. Heat Mass Transf..

[26] Osara J.A., Bryant M.D. (2019). Thermodynamics of Lead-Acid Battery Degradation: Application of the Degradation-Entropy Generation Methodology. J. Electrochem. Soc..

[27] Osara J.A., Bryant M.D. (2024). Methods to Calculate Entropy Generation. Entropy.

[28] Bryant M.D., Khonsari M.M., Ling F.F. (2008). On the Thermodynamics of Degradation. Proc. R. Soc. Math. Phys. Eng. Sci..

[29] Moran M.J., Shapiro H.N. (2004). Fundamentals of Engineering Thermodynamics.

[30] Kondepudi D., Prigogine I. (1998). Modern Thermodynamics from Heat Engines to Dissipative Structures.

[31] Osara J.A., Ezekoye O.A., Marr K.C., Bryant M.D. (2021). A Methodology for Analyzing Aging and Performance of Lithium-Ion Batteries: Consistent Cycling Application. J. Energy Storage.

[32] Burghardt M.D., Harbach J.A. (1993). Engineering Thermodynamics.

[33] Çengel Y.A. (2008). Thermodynamics: An Engineering Approach.

[34] Kuhn E. (2015). Correlation between System Entropy and Structural Changes in Lubricating Grease. Lubricants.

[35] Lugt P.M. (2013). Grease Lubrication in Rolling Bearings.

[36] Rincon J.S., Osara J., Bryant M.D., Fernández B.R. Shannon’s Machine Capacity & Degradation-Degradation-Entropy Generation Methodologies for Failure Detection: Practical Applications to Motor-Pump Systems. Proceedings of the 37th International Pump Users Symposium; Turbomachinery Laboratory, Texas A&M Engineering Experiment Station.

[37] Bryant M.D., Osara J.A. (2024). On Degradation-Entropy Generation Theorems and Vector Spaces for Irreversible Thermodynamics. Appl. Mech..

[38] Feng X., Ouyang M., Liu X., Lu L., Xia Y., He X. (2018). Thermal Runaway Mechanism of Lithium Ion Battery for Electric Vehicles: A Review. Energy Storage Mater..

[39] Ouyang M., Ren D., Lu L., Li J., Feng X., Han X., Liu G. (2015). Overcharge-Induced Capacity Fading Analysis for Large Format Lithium-Ion Batteries with LiyNi_1_/_3_Co_1_/_3_Mn_1_/_3_O_2_ + LiyMn_2_O_4_ Composite Cathode. J. Power Sources.

[40] Rohatgi A. WebPlotDigitizer. Version 4.8. https://apps.automeris.io/wpd4/.

[41] Bejan A., Kestin J. (1983). Entropy Generation through Heat and Fluid Flow. J. Appl. Mech..

